# Nonequilibrium Dynamics of a Magnetic Nanocapsule in a Nematic Liquid Crystal

**DOI:** 10.3390/ma14112886

**Published:** 2021-05-27

**Authors:** José Armendáriz, Humberto Híjar

**Affiliations:** Engineering of School, La Salle University Mexico, Benjamin Franklin 45, Mexico City 06140, Mexico; jose.armendariz@lasallistas.org.mx

**Keywords:** nematic colloids, topological defects, rotational Brownian motion, multiscale simulation methods, 42.70.Df, 82.70.Dd, 61.30.Jf, 05.70.Ln

## Abstract

Colloidal particles in nematic liquid crystals show a beautiful variety of complex phenomena with promising applications. Their dynamical behaviour is determined by topology and interactions with the liquid crystal and external fields. Here, a nematic magnetic nanocapsule reoriented periodically by time-varying magnetic fields is studied using numerical simulations. The approach combines Molecular Dynamics to resolve solute–solvent interactions and Nematic Multiparticle Collision Dynamics to incorporate nematohydrodynamic fields and fluctuations. A Saturn ring defect resulting from homeotropic anchoring conditions surrounds the capsule and rotates together with it. Magnetically induced rotations of the capsule can produce transformations of this topological defect, which changes from a disclination curve to a defect structure extending over the surface of the capsule. Transformations occur for large magnetic fields. At moderate fields, elastic torques prevent changes of the topological defect by tilting the capsule out from the rotation plane of the magnetic field.

## 1. Introduction

Nematic liquid crystals are anisotropic fluids usually formed of rodlike molecules that exhibit no positional order but self-organised orientational order [1]. The order parameter *S* and the so-called director field $\hat{n}$ describe the degree of order and the average molecular orientation of the nematic state, respectively. When doped with colloidal particles (CPs), nematic liquid crystals become enormously interesting due to fundamental and technological reasons [2,3,4]. CPs induce strong deformations on $\hat{n}$ that depend on the colloidal shape and size and on the anchoring conditions that colloidal surfaces impose on liquid crystal molecules [5]. The interplay between long-range orientational order and superficial alignment inevitably creates topological defects around the CP-regions where molecular ordering is frustrated and *S* is strongly depressed. The typical radius of these defects is $r_{c}≃10\;\mathrm{nm}$ [6]. However, director deformations extend to cover micrometric scales creating long-range interactions that are exclusive for CPs dispersed in nematic solvents [7]. This promotes self-assembly of CPs and facilitates the fabrication of composite structures with novel designed optical and mechanical properties [8].

Early investigations on nematic colloids focused on spherical solutes [9,10]. Recently, advances in fabrication techniques of CPs have permitted extending the study to a huge variety of nonspherical micro- and nanosized particles. Examples include rodlike, ellipsoidal, and spherocylindrical CPs [11,12,13,14]; bullet and peanut-shaped CPs [15,16]; and polygonal platelets, handlebodies, and Koch fractal microparticles [17,18,19,20]. Through these studies, defects and interactions generated by anisotropic particles in nematic liquid crystals in thermodynamic equilibrium have been explored.

To a lesser degree, the effects of driving nematic colloids away from equilibrium have also been investigated. Under specific conditions regarding size, confinement, and anchoring strength, microscopic spheres with homeotropic anchoring, for which $\hat{n}$ is perpendicular to the colloidal surface, are surrounded by ringlike topological defects known as Saturn-rings [5,21,22]. Flow past these CPs distorts and moves defect rings along the relative flow [23,24,25,26,27,28,29]. In the limit of vanishing inertial contributions, rings around nanospheres move upstream and collapse into a point defect of the hedgehog type [30]. Effects of flow could be used as means for particle selection [24] or self-assembly control [30].

The motion of CPs in nematic liquid crystals can be controlled by micropost arrays [31,32], patterned substrates [33], or undulating walls [34,35]. The latter produce gradients of the director field that attract CPs to particular locations of minimum elastic energy, thus giving rise to a lock-and-key mechanism. As spherical and ellipsoidal CPs move towards the wavy walls, their accompanying Saturn-rings are displaced contrary to the motion of the CPs and suffer a transition to a dipole defect induced by the elastic energy landscape [34]. Morphological changes of topological defects have been also reported for spherocylindrical CPs in a nematic host phase under shear flow [36]. In this case, the relative rotation between $\hat{n}$ and the CP induces hydrodynamic torques on the former, which periodically broaden and extend the accompanying ring defect over the cylindrical portion of the CP. The high elastic energy associated with this transformation is paid by the externally imposed shear flow.

Optical tweezers, provide important additional degrees of freedom to control the motion of microparticles in liquid crystals [37,38], whereas magnetic fields can be used for colloid-molecule coupling and rotatory manipulation [39,40]. A delicately adjusted relation between fluid-imposed, magnetic, and gravitational forces has been used for levitating magnetic nanowires in nematic liquid crystals [41]. Magneto-optic manipulations permit generation of nontrivial topological defects around individual spherical CPs and small colloidal chains [42]. The effect of a rotatory magnetic field on a peanut-shaped CP with a magnetic moment perpendicular to its long symmetry axis has been investigated [16]. The CP rotates as the magnetic field does, while the director field at far distances is unperturbed since the diamagnetic anisotropy of the liquid crystal is very small. The combined effect of magnetic and elastic torques on these CPs is used to estimate their magnetic dipole moment and to control their self-assembly along small chains [16]. Understanding the dynamics of anisotropic nematic colloids under magnetic fields is an important scientific challenge with additional applications in the use of nanoparticles for tuning the Freedericksz transition threshold and orientation relaxation times in liquid crystal cells [43].

Here, magnetically induced rotations of an anisotropic CP in a nematic liquid crystal are studied. The CP is considered to impose homeotropic anchoring conditions. This problem is analysed from a numerical perspective based on a hybrid algorithm combining Molecular Dynamics (MD) and Nematic Multiparticle Collision Dynamics (N-MPCD) [29,36]. This allows us to simulate the nematohydrodynamic behaviour of the solvent and the dynamics of the topological defects surrounding the rotating CP, which are found to be a disclination for bent rings. In addition, the numerical approach allows us to vary relevant parameters, including the frequency and strength of the magnetic field and the order parameter of the solvent, over ranges that permit identification of different rotatory regimes departed from equilibrium. The analysis is restricted to time-varying magnetic fields B(t) that rotate at small and moderate frequencies on a plane containing the director field at far distances from the CP ${\hat{n}}_{0}$. At small frequencies, the CP and its defect ring simultaneously follow the rotation of B. At higher frequencies, rotation of the CP is retarded with respect to B by a measurable time lapse. This behaviour is modelled in terms of a stochastic linear equation of the Langevin-type, from which relaxation of the CP towards the instantaneous equilibrium state dictated by B is analysed and a good agreement with numerical results is found. As the capsule rotates at small magnetic fields, it is also inclined out from the ${\hat{n}}_{0}-B$ plane when its long symmetry axis approaches ${\hat{n}}_{0}$ to avoid large increments of the elastic energy. For large fields, the companion defect changes its form from an elongated Saturn ring to an extended structure that surrounds most of the surface of the capsule. This transformation occurs as an attempt of the solvent to release the elastic energy accumulated, as capsule and defect are driven through nonequilibrium configurations. Defect transformations are present for small rotational Reynolds numbers Re(r)∼10−2 and small Ericksen numbers $\mathrm{Er}∼10^{-2}$, suggesting that they could be experimentally reproduced.

## 2. Methods

**Figure 1:**
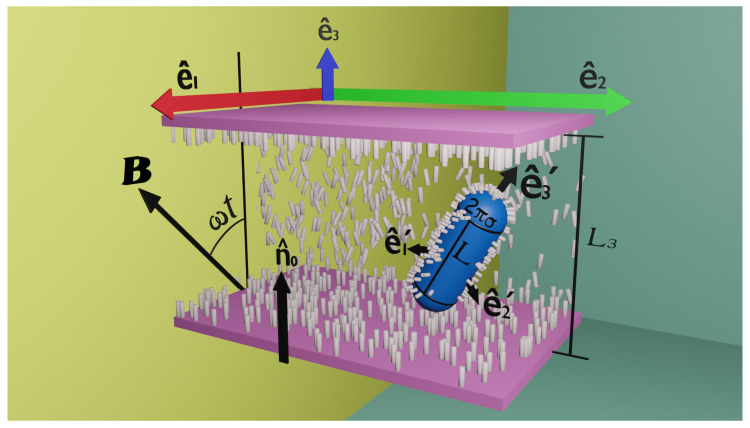
Scheme of the simulated system showing a spherocylindrical capsule in a nematic cell. Small bars indicate the local director field. The perimeter of the cylindrical part of the CP is 2πσ, as indicated. The capsule has a permanent magnetic moment in the direction of ${\hat{e}}_{3}^{\prime }$ and its surface imposes homeotropic anchoring on the director field.

A magnetic nanocapsule with spherocylindrical shape is placed into a nematic cell of thickness $L_{3}$ and homeotropic anchoring conditions, as depicted in Figure 1, where *L* and σ indicate the length of the cylindrical portion and the radius of the capsule, respectively. Two auxiliary frames are used to describe the system. The first one is a laboratory frame spanned by unit vectors $\left\{{\hat{e}}_{1},{\hat{e}}_{2},{\hat{e}}_{3}\right\}$. The second one is attached to the nanocapsule and is spanned by vectors $\left\{{\hat{e}}_{1}^{\prime },{\hat{e}}_{2}^{\prime },{\hat{e}}_{3}^{\prime }\right\}$, where ${\hat{e}}_{3}^{\prime }$ coincides with the long symmetry axis. Hereafter, the orientation of the capsule will be specified by the angle $\theta \left(t\right)=\cos^{-1}\left({\hat{e}}_{3}^{\prime }\left(t\right)\cdot {\hat{e}}_{3}\right)$. The magnetic moment of the CP is $\mu =\mu {\hat{e}}_{3}^{\prime }\left(t\right)$, and the external magnetic field changes periodically according to $B\left(t\right)=B\left[\sin\left(\omega_{0}t\right){\hat{e}}_{1}+\cos\left(\omega_{0}t\right){\hat{e}}_{3}\right]$. For illustrative purposes only, small elongated particles are used in Figure 1 to represent the director field within the cell. The capsule promotes homeotropic anchoring on its surface, whereas anchoring on the confining plates imposes the condition ${\hat{n}}_{0}={\hat{e}}_{3}$. It is assumed that the diamagnetic anisotropy of the solvent is very small. Thus, B(t) does not have any effect on the orientation of the director.

To study the response of the CP to B(t), numerical simulations are implemented. The employed algorithm has a twofold scope. It integrates Newton’s laws of motion of the solvent–solute system over a small time-step and incorporates slower hydrodynamic modes of the solvent by mimicking collective interactions between its microscopic degrees of freedom. This approach has been successfully used to simulate topological defects around spherical and spherocylindrical nematic colloids in equilibrium and nonequilibrium conditions imposed by flow [29,36]. It also reproduces equilibrium states of CPs under static magnetic fields [44]. For self-containment, and considering that the method is rather novel, complete details of the algorithm are given below.

### 2.1. Molecular Dynamics

On the perspective of the mesoscopic method to be used at the large time-scale, the solvent is simulated by *N* point particles carrying a unit orientation vector. Positions, velocities, and orientations of solvent particles are taken as continuous functions of *t* and denoted as $r_{i}$, $v_{i}$, and ${\hat{u}}_{i}$, respectively, for i=1,2,…,N. These particles interact with the capsule through a repulsive potential [45],

(1) $$\Phi \left(r_{i}-R\right)=\left\{\begin{array}{cc} 4\epsilon \left[{\left(\frac{\sigma }{\ell_{i}}\right)}^{48}-{\left(\frac{\sigma }{\ell_{i}}\right)}^{24}+\frac{1}{4}\right], & \mathrm{if}\ell_{i}\leq 2^{1/24}\sigma \;\\ 0, & \mathrm{otherwise} \end{array}\right.,$$

where ϵ is the interaction strength and $\ell_{i}$ is the minimum distance from $r_{i}$ to the line segment defining the longitudinal symmetry axis of the cylindrical portion of the capsule.

Forces on solvent and solute particles are given by $f_{i}=-\nabla \Phi$ and $F=-\sum_{i=1}^{N}f_{i}$, respectively. The capsule also experiences a total torque $k=\mu \times B-\sum_{i=1}^{N}\left(r_{i}-R\right)\times f_{i}$, where R is the centre-of-mass position of the CP. The equations of motion of solvent particles are resolved over a small time-step δt according to the MD velocity-Verlet algor ithm [46]

(2) $$r_{i}\left(t+\delta \;t\right)\;=\;r_{i}\left(t\right)+v_{i}\left(t\right)\delta \;t+\frac{1}{2m}f_{i}\left(t\right){\left(\delta \;t\right)}^{2},$$

(3) $$v_{i}\left(t+\delta \;t\right)\;=\;v_{i}\left(t\right)+\frac{f_{i}\left(t\right)+f_{i}\left(t+\delta \;t\right)}{2m}\delta \;t,$$

where *m* is the mass of the solvent particles. Similar equations are used to integrate R and the centre of mass velocity of the capsule, whereas its rotational dynamics is integrated in terms of the transformation matrix between the laboratory and moving coordinate systems A and the angular velocity and angular momentum of the CP, Ω and l, respectively. The former satisfies ${\hat{e}}_{\alpha }^{\prime }=A\cdot {\hat{e}}_{\alpha }$, and is updated according to the second order expansion

(4) $$A\left(t+\delta \;t\right)=A\left(t\right)+\dot{A}\left(t\right)\delta \;t+\frac{1}{2}\ddot{A}\left(t\right){\left(\delta \;t\right)}^{2}.$$

It has been shown in reference [47] that time derivatives of A and Ω are related through $\dot{A}=W\cdot A$, and $\ddot{A}=\dot{W}\cdot A+W\cdot W\cdot A$, where

(5) $$W=\left(\begin{array}{ccc} 0 & \Omega_{3} & -\Omega_{2}\\ -\Omega_{3} & 0 & \Omega_{1}\\ \Omega_{2} & -\Omega_{1} & 0 \end{array}\right).$$

This permits use of the Euler equations

(6) $${\dot{\Omega }}_{1}=\frac{1}{J_{1}}\left[\kappa_{1}\left(t\right)+\left(J_{2}-J_{3}\right)\Omega_{2}\left(t\right)\Omega_{3}\left(t\right)\right],$$

(7) $${\dot{\Omega }}_{2}=\frac{1}{J_{2}}\left[\kappa_{2}\left(t\right)+\left(J_{3}-J_{1}\right)\Omega_{3}\left(t\right)\Omega_{1}\left(t\right)\right],$$

(8) $${\dot{\Omega }}_{3}=\frac{1}{J_{3}}\left[\kappa_{3}\left(t\right)+\left(J_{1}-J_{2}\right)\Omega_{1}\left(t\right)\Omega_{2}\left(t\right)\right],$$

to calculate all terms on the right-hand side of Equation (4). In Equations (6)–(8), $J_{1}$, $J_{2}$, and $J_{3}$ are the principal moments of inertia of the capsule ($J_{1}=J_{2}$); and κ=A·k. Notice that to sustain the evolution of A, a rule for updating Ω is also required. This is given indirectly by writing Ω in terms of the angular momentum in the laboratory system, $\Omega =J\cdot A^{-1}\cdot l$, and using a velocity-Verlet rule to update l, namely,

(9) $$l\left(t+\delta \;t\right)=l\left(t\right)+\frac{k(t)+k(t+\delta \;t)}{2}\delta \;t.$$

### 2.2. Nematic Multiparticle Collision Dynamics

The mesoscopic behaviour of the solvent will be simulated through a coarse-grained approach that permits reproducing the density, orientation, and flow fields around the CP. This approach is inspired in Multiparticle Collision Dynamics (MPCD), a very useful technique for the simulation of diverse systems in soft condensed matter, including colloids and polymer solutions in isotropic liquids [48,49]. MPCD has been recently generalised to nematic liquid crystals [50,51,52]. The approach used in this paper corresponds to the specific method proposed by Shendruk and Yeomans in reference [51], in which collective collisions between solvent particles take place in localised space regions at regular time intervals of size Δt≫δt. These collisions are conducted by stochastic collision operators that modify orientations and velocities of the solvent particles as described below.

To apply such operators, the simulation box is subdivided into adjacent cubic regions with volume $a^{3}$, hereafter referred to as collision cells. The number of solvent particles within each collision cell $N^{c}\left(t\right)$ is different and changes with time. Particles in the same cell are used to approximate mesoscopic fields such as velocity, order parameter, and director, $v^{c}$, $S^{c}$, and $n^{c}$, respectively. The former is defined as

(10) $$v^{c}=\frac{1}{N^{c}\left(t\right)}\sum_{j=1}^{N^{c}}v_{j},$$

while $S^{c}$ and $n^{c}$ are estimated as the largest eigenvalue and the corresponding eigenvector of the order parameter tensor [45]

(11) $$Q^{c}=\frac{1}{2N^{c}\left(t\right)}\sum_{j=1}^{N^{c}}\left(3{\hat{u}}_{j}{\hat{u}}_{j}-I\right),$$

where I is the identity matrix.

Some other auxiliary fields that are also calculated are the centre of mass position

(12) $$r^{c}=\frac{1}{N^{c}\left(t\right)}\sum_{j=1}^{N^{c}}r_{j},$$

the inertia tensor

(13) $$J^{c}=m\sum_{j=1}^{c}\left[{\left(r_{j}-r^{c}\right)}^{2}I-\left(r_{j}-r^{c}\right)\left(r_{j}-r^{c}\right)\right],$$

and the velocity and orientation gradients $\nabla v^{c}$ and $\nabla n^{c}$, respectively, which are approximated from finite differences of fields $v^{c}$ and $n^{c}$ over the grid of collision cells.

N-MPCD considers a mean-field interaction between solvent particles located in the same collision cell. Specifically, the Maier–Saupe potential [53]

(14) $$U\left({\hat{u}}_{i}\right)=-\frac{3}{2}US^{c}\left[{\left({\hat{u}}_{i}\cdot {\hat{n}}^{c}\right)}^{2}-\frac{1}{3}\right]$$

quantifies the tendency of orientations ${\hat{u}}_{i}$ to align with the director produced by all particles located in the same cell ${\hat{n}}^{c}$. Parameter *U* in Equation (14) tunes the strength of the mean-field potential and is referred to as the nematicity of the solvent. Shendruck and Yeomans have shown that N-MPCD fluids exhibit nematic behaviour for $U≳5k_{B}T$, with the Boltzmann constant $k_{B}$ and temperature *T*, and that they exhibit an isotropic phase otherwise. The stochastic collision operator for orientations takes new vectors ${\hat{u}}_{i}$ from the canonical distribution $Z^{-1}\exp\left(-U\left({\hat{u}}_{i}\right)/k_{B}T\right)$, where $Z^{-1}$ is a normalisation constant.

In turn, the collision operator for velocities samples new $v_{i}$ vectors from the Maxwell velocity distribution using the Andersen thermostat [48]

(15) $$v_{i}=v^{c}+\xi_{i}-\xi^{c}-\left[{\left(J^{c}\right)}^{-1}\cdot \Delta \;L^{c}\right]\times \left(r_{i}-r^{c}\right),$$

where $\xi_{i}$ is the randomly sampled velocity $\xi^{c}=\sum_{j=1}^{N^{c}}\xi_{j}$ and $\Delta \;L^{c}$ is the angular momentum generated by the velocity change. The third and fourth terms on the right-hand side of Equation (15) guarantee conservation of linear and angular momentum, respectively.

An important feature of nematic liquid crystals is that director and flow fields are strongly coupled. Nonuniform flow induces director’s reorientation, which might cause hydrodynamic motion known as backflow [1]. N-MPCD cares about this issue through the implementation of rules that recreate the two-way coupling. To simulate reorientation by flow, solvent particles are considered to behave as slender rods with tumbling parameter λ. Orientation changes over the time interval Δt are given by Jeffery’s equation [54]:

(16) $$\Delta \;{\hat{u}}_{i}=\chi_{\mathrm{HI}}\left[C^{c}\cdot {\hat{u}}_{i}-\lambda \;\left(D^{c}\cdot {\hat{u}}_{i}-D^{c}\cdot {\hat{u}}_{i}{\hat{u}}_{i}{\hat{u}}_{i}\right)\right]\Delta \;t,$$

where $C^{c}$ and $D^{c}$ are the vorticity and strain rate matrices, i.e., the antisymmetric and symmetric parts of $\nabla v^{c}$, respectively. Moreover, $\chi_{\mathrm{HI}}$ is a control parameter restricted to the interval [0,1] that tunes the relaxation time for solvent particles relative to Δt. The dynamic response of the director field in the presence of flow is dictated by λ. For λ≲1, nematic particles perform a continuous rotation known as tumbling. In contrast, for λ≳1, a static alignment under flow is achieved [51].

Backflow is taken into account by translating the angular momentum generated by reorientation of solvent particles within each collision cell $\Delta \;L_{\mathrm{ori}}^{c}$ into linear momentum. With this aim, the reorientation process is considered to be overdamped; thus, $\Delta \;L_{\mathrm{ori}}^{c}$ can be written in the form

(17) $$\Delta \;L_{\mathrm{ori}}^{c}=\gamma_{R}\sum_{j=1}^{N^{c}}{\hat{u}}_{j}\left(t+\Delta \;t\right)\;\times \;{\hat{u}}_{j}\left(t\right),$$

where $\gamma_{R}$ is a heuristic viscous rotation coefficient and ${\hat{u}}_{j}\left(t+\Delta \;t\right)$ and ${\hat{u}}_{j}\left(t\right)$ correspond to orientations after and before the N-MPCD collision event, respectively. $\Delta \;L_{\mathrm{ori}}^{c}$ is transferred as part of $\Delta \;L^{c}$ in Equation (15). It is worth noticing that other versions of N-MPCD resolve backflow differently. In particular, they use a method based on theoretical formulations of nematohydrodynamics [50,52].

To quantify the elastic energy stored in the nematic medium, the Frank free energy density is considered under the one-constant approximation, which has been proven to be valid for the present implementation of N-MPCD [36,51,55]. The total elastic energy of the nematic solvent is

(18) $$F_{\mathrm{elas}}=a^{3}\frac{K}{2}\sum_{c}\left[{\left(\nabla \cdot {\hat{n}}^{c}\right)}^{2}+{\left(\nabla \times {\hat{n}}^{c}\right)}^{2}\right],$$

where *K* is the elastic constant and $\sum_{c}$ indicates summation over all collision cells. By using Equation (18), it is assumed that defect cores are not fully resolved at the coarse-grained scale of N-MPCD. Thus, ${\hat{n}}^{c}$ presents no singularities and its spatial derivatives are well-defined.

### 2.3. Boundary and Anchoring Conditions

Confinement is simulated by imposing bounce-back boundary conditions, which completely reverse the velocity of particles encountering solid walls at $x_{3}=0$ and $x_{3}=L_{3}$, while periodic boundary conditions are implemented along ${\hat{e}}_{1}$ and ${\hat{e}}_{2}$. As it is customary in MPCD methods, the simulation box suffers a random displacement along a vector with components in the range [0,a], where prior collisions take place. This is intended to preserve Galilean invariance in the system [56]. Due to bounce-back boundary conditions, empty spaces appear after this random displacement and virtual particles can be used to fill them. Virtual particles have the same mass and degrees of freedom as solvent particles and are allowed to participate during the momentum exchange event. To this aim, they are inserted randomly within two additional layers at the top and bottom of the simulation box with the same numerical density as the nematic fluid and velocities sampled from the Maxwell distribution at temperature *T*. The centre of mass velocity in the additional layers is cancelled. These conditions guarantee that momentum flux at the boundary will be correctly simulated during the multiparticle velocity exchange step [57]. Furthermore, virtual particles can be used to control anchoring conditions on the confining surfaces. Orientations of all virtual particles are chosen along ${\hat{e}}_{3}$. This favours the alignment of ${\hat{n}}^{c}$ along the $x_{3}$ axis in collision cells containing virtual particles, since they are also allowed to contribute to the orientation exchange operator. Thus, their inclusion promotes effective homeotropic anchoring because ${\hat{u}}_{i}≃{\hat{e}}_{3}$ for particles close to $x_{3}=0$ and $x_{3}=L_{3}$.

Similarly, the solvent experiences homeotropic anchoring conditions over the surface of the magnetic capsule. These are implemented with the aid of two mechanisms. First, nematic particles that satisfy the condition $\ell_{i}\leq 2^{1/24}\sigma$ during the velocity-Verlet step—see Equation (1)—are reoriented along the perpendicular direction to the surface of the capsule at $r_{i}\left(t\right)$. Second, an additional set of massless virtual particles is attached at the surface of the CP, all of which point along the normal direction to its surface. They are randomly distributed over this surface with a superficial numerical density $\sigma_{S}$ and reorient solvent particles in their vicinity analogously as virtual particles used for the confining walls. It has been shown that these two mechanisms promote strong homeotropic anchoring on the colloidal surface and permit the formation of topological defects around the capsule for equilibrium and nonequilibrium flow conditions [36].

### 2.4. Analytical Description of Capsule Rotation

The analysis in this paper is supplemented by a simplified dynamical model that permits validation of the simulation method. In this model, the magnetic CP is assumed to perform rotations on the ${\hat{e}}_{1}-{\hat{e}}_{3}$ plane while it follows B(t). The magnetic potential is $F_{\mathrm{mag}}=-\mu B\cos\vartheta$, where ϑ is the angle between ${\hat{e}}_{3}^{\prime }$ and B(t). The nematic environment imposes an effective potential on the CP, which can be approximated as $F_{\mathrm{nem}}=F_{⊥}+\left(F_{\|}-F_{⊥}\right)\cos^{2}\theta$, where $F_{\|}$ and $F_{⊥}$ are configuration energies achieved at parallel and perpendicular orientations of the CP with respect to ${\hat{n}}_{0}$, θ=0 and θ=π/2, respectively. This specific form for $F_{\mathrm{nem}}$ was originally proposed by Burylov and Raikher in Reference [58] and explains the tendency of elongated particles to achieve perpendicular orientation with respect to ${\hat{n}}_{0}$ for $F_{\|}-F_{⊥}=\Delta \;F>0$. The capsule’s orientation is considered to obey the equation of motion

(19) $$J_{1}\ddot{\theta }=-\eta_{R}\dot{\theta }-\frac{d}{d\theta }\left(F_{\mathrm{mag}}+F_{\mathrm{nem}}\right)\;+\;\tau \left(t\right),$$

where $\eta_{R}$ is the rotational friction coefficient and τ(t) is a stochastic torque. The latter is assumed to be a zero average Gaussian–Markov process with

(20) $$\langle \tau \left(t^{\prime }\right)\tau \left(t\right)\rangle =2\eta_{R}k_{B}T\delta \left(t^{\prime }-t\right),$$

where brackets indicate stochastic average and $\delta (t^{\prime }-t)$ denotes Dirac delta function.

As it is shown in Appendix A, in the limit of large magnetic torques, the average stationary rotation of the capsule is retarded from B(t) and slightly deviated from pure sinusoidal dependence according to

(21) $$\theta_{\mathrm{stat}}\left(t\right)=\omega_{0}t-\phi_{\theta }+\frac{\Delta \;F}{{\left[{\left(\mu B-2\omega_{0}^{2}J_{1}\right)}^{2}+4\omega_{0}^{2}\eta_{R}^{2}\right]}^{1/2}}\sin\left(2\omega_{0}t+\phi_{\vartheta }\right),$$

where the phase retardation is $\phi_{\theta }=\eta_{R}\omega_{0}/\left(\mu B\right)$ and $\phi_{\vartheta }=\tan^{-1}\left(-2\omega_{0}\eta_{R}/\left(\mu B-4\omega_{0}^{2}J_{1}\right)\right)$.

Thermal noise around the main orientation can be described in terms of ϑ, representing the angle between ${\hat{e}}_{3}^{\prime }$ and the instantaneous magnetic field. In Appendix A, it is shown that the normalised self-correlation function of ϑ in Fourier domain can be approximated as

(22) $$\chi \left(\omega \right)=\frac{\langle \vartheta \left(\omega \right)\vartheta^{*}\left(\omega \right)\rangle }{\langle \vartheta \left(0\right)\vartheta^{*}\left(0\right)\rangle }=\frac{\omega_{B}^{4}}{\left[{\left(\omega \;-\beta_{1}\right)}^{2}+\beta^{2}\right]\left[{\left(\omega \;+\beta_{1}\right)}^{2}+\beta^{2}\right]},$$

where $\omega_{B}$ and β are defined in Appendix A and $\beta_{1}=J_{1}^{-1}\sqrt{{\left(\mu B\right)}^{2}-\eta_{R}^{2}/4}$.

The model summarised by Equations (21) and (22) show a good agreement with simulations based on the MD-N-MPCD algorithm in the correct limit.

## 3. Results

### 3.1. Simulation Parameters

In this paper, results are presented in simulation units using *a*, *m*, and $k_{B}T$ as units of length, mass, and energy, respectively (this corresponds to fixing a=1, $k_{B}T=1$, and m=1 in the simulation code) [49]. All other mechanical quantities have units derived from the previous ones. Table 1 summarises these derived units as well as the parameters used during the simulation stage. In Table 1, $t_{0}$ and $\eta_{0}$ indicate units of time and viscosity, respectively; ${\bar{N}}^{c}$ is the average numerical density of the solvent (number of N-MPCD particles per collision cell); $\rho_{\mathrm{col}}$ is the mass density of the magnetic capsule, which is set five times larger than the solvent’s density. Concerning the magnetic field, four different strengths were used. In terms of the magnetic energy, they are expressed as μB=0, 10, 20, and $50\;k_{B}T$. Two rotation frequencies were considered, namely, $\omega_{0}=\pi \times 10^{-4}t_{0}^{-1}$ and $2\pi \times 10^{-4}t_{0}^{-1}$.

The size of the simulated capsule could be estimated in terms of the defect core radius, which, for N-MPCD, is found to be $r_{c}≃a$ [29]; thus, *L* is scaled to L≃100nm.

The shear viscosity of the simulated solvents η can be calculated from the fluid parameters listed in Table 1 and the well known formulae of MPCD analytical descriptions. Specifically [48],

(23) $$\eta \;=k_{B}T{\bar{N}}^{c}\Delta \;t\left(\frac{a^{3}{\bar{N}}^{c}}{a^{3}{\bar{N}}^{c}-\frac{5}{4}}-\frac{1}{2}\right)+\frac{ma^{2}{\bar{N}}^{c}}{24\Delta \;t}\left(1-\frac{7}{5a^{3}{\bar{N}}^{c}}\right),$$

which yields $\eta \;=12.11\eta_{0}$. It is worth noticing that due to their anisotropic character, actual nematic solvents have more than one viscosity coefficient, whereas N-MPCD approximates the momentum transport through the solvent as isotropic [55]. However, this approximation has shown consistent results for simulations of CPs driven by nematic flow [29,36] and can be considered as a first attempt to reproduce the hydrodynamic behaviour on the mesoscopic scale.

The selection of tumbling parameter λ=1.2 will produce solvents with shear alignment behaviour. Notice that in Table 1, two nematicities are given $U=6.5\;k_{B}T$ and $10\;k_{B}T$, which yield simulations for two distinct solvents with average order parameters ${\bar{S}}^{c}=0.60$ and 0.74, respectively. The elastic coefficients for these solvents are also different. They can be estimated from order parameter fluctuations, as described in [36,51]. This yields $K≃151\;k_{B}T/a$ and $K≃232\;k_{B}T/a$, for $U=6.5\;k_{B}T$ and $U=10\;k_{B}T$, respectively.

All simulations were executed for a total time interval $2.5\times 10^{4}\;t_{0}$, and a thermalisation stage lasting $0.5\times 10^{4}\;t_{0}$ is considered.

### 3.2. Capsule’s Rotatory Motion

**Figure 2:**
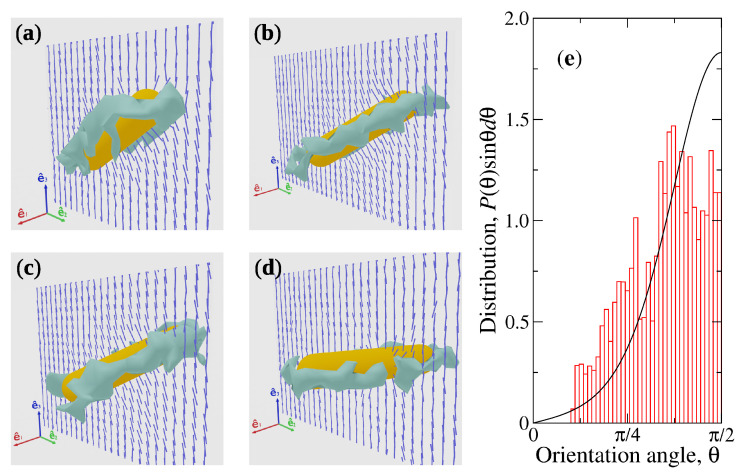
The capsule’s orientation in the absence of magnetic field. (**a**–**d**) Snapshots of the CP at regular time intervals showing the director field around it as small line segments and the topological defect as an isosurface for order parameter $S^{c}=0.5$. (**e**) Probability distribution function for orientation with respect to ${\hat{n}}_{0}$. The histogram corresponds to simulation results and the continuous curve to a fit based on the canonical distribution.

The simulated CP performs translational and rotational Brownian motion when immersed in the N-MPCD fluid. A topological defect in the form of an elongated Saturn ring appears in the vicinity of the CP, as illustrated in Figure 2a–d, which are four snapshots of the nematic-capsule system taken at regular time intervals after the thermalisation stage of simulations with B=0. Disclination rings are bent in opposite directions close to the extremes of the CP and are essentially flat when the latter has perpendicular orientation relative to ${\hat{n}}_{0}$, θ=π/2. Supplementary Video S1 provides an animation of the motion of the capsule at B=0. In the absence of magnetic field, configurations close to perpendicular are visited more frequently than others. This indicates that ${\hat{e}}_{3}^{\prime }⊥{\hat{n}}_{0}$ is the most stable state for the capsule and ΔF>0. To estimate ΔF precisely, the numerical probability distribution of θ is obtained and compared with the canonical distribution $P\left(\theta \right)\sin\theta \;d\theta =\exp\left(-\Delta \;F\cos^{2}\theta \;/k_{B}T\right)\sin\theta \;d\theta /Z$, where Z is the partition function. By considering ΔF as an adjustable parameter, values $\Delta \;F=1.75\;k_{B}T$ and $2.5\;k_{B}T$ are obtained for $U=6.5\;k_{B}T$ and $10\;k_{B}T$, respectively. As expected, the capsule’s orientations is closer to the ${\hat{e}}_{1}-{\hat{e}}_{2}$ plane at larger nematicity. Figure 2e shows the comparison between the numerical and canonical orientation distributions.

**Figure 3:**
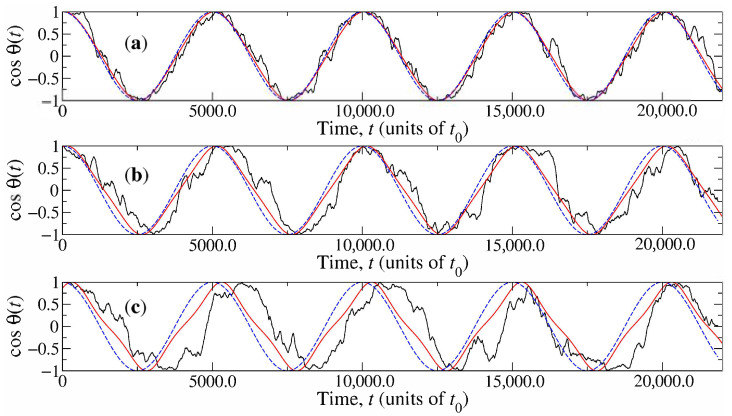
Angular displacement of the magnetic capsule under a rotatory magnetic field. Noisy and continuous curves represent, respectively, simulation results and the approximation given by Equation (21). Dashed curves indicate the instantaneous projection of the magnetic field along ${\hat{e}}_{3}$. (**a**) $\mu B=50\;k_{B}T$; (**b**) $\mu B=20\;k_{B}T$; (**c**) $\mu B=10\;k_{B}T$.

The capsule’s rotations are induced when the magnetic field is applied. These take place on the ${\hat{e}}_{1}-{\hat{e}}_{3}$ plane, though deviations outside this plane can be observed and become appreciable mainly in the case $\mu B=10\;k_{B}T$. The dynamic orientation of the nanocapsule is represented through the function cosθ(t) in Figure 3, where the noisy trajectory indicates the actual time series observed in simulations with $\omega_{0}=2\pi \times 10^{-4}t_{0}^{-1}$ and the continuous curve is the approximation $\cos(\theta_{\mathrm{stat}}\left(t\right))$, obtained from the model in Section 2.4. For comparative purposes, the function $B_{3}\left(t\right)/B=\cos\left(\omega_{0}t\right)$ has been included in Figure 3 as a dashed curve representing the rotation of B. To calculate the stationary solution $\theta_{\mathrm{stat}}\left(t\right)$ in Equation (21), it is considered that the rotational friction coefficient of the capsule is similar to that presented by a long rod of length *L* and radius σ [59]:

(24) $$\eta_{R}=b\frac{\pi L^{3}}{4+3\ln\left(1+\frac{L}{2\sigma }\right)}\eta ,$$

where *b* is a factor that can be adjusted to correct specific differences in slip boundary conditions and shape. It has been estimated to be b≃0.6 [36,44]. Figure 3 exhibits a good agreement between simulations and theory. For large magnetic fields ($\mu B=50\;k_{B}T$, in Figure 3a), the model predicts no appreciable difference between $\theta_{\mathrm{stat}}\left(t\right)$ and $\omega_{0}t$. Correspondingly, the capsule follows the magnetic field very closely, performing small thermal fluctuations around the main trajectory. The phase retardation between $\cos(\theta_{\mathrm{stat}}\left(t\right))$ and $B_{3}\left(t\right)/B$ predicted by the model becomes noticeable for moderate fields ($\mu B=20\;k_{B}T$, Figure 3a). This retardation is present in simulations and can be appreciated since, in Figure 3b, the noisy trajectory is closer to the continuous curve than to the dashed curve. Notice also that fluctuations in the case $\mu B=20\;k_{B}T$ are stronger than those in the case $\mu B=50\;k_{B}T$. These effects are more pronounced for $\mu B=10\;k_{B}T$ (Figure 3c), where retardation in simulations is larger than that predicted analytically. This can be explained by noticing that stronger thermal fluctuation reduce the average projection of ${\hat{e}}_{3}^{\prime }$ on the ${\hat{e}}_{1}-{\hat{e}}_{3}$ plane. Thus, the effective magnetic torque along ${\hat{e}}_{2}$ decreases from the value assumed in the two-dimensional linear model, μB, which, in turn, produces an increment of $\phi_{\theta }$ in Equation (21).

**Figure 4:**
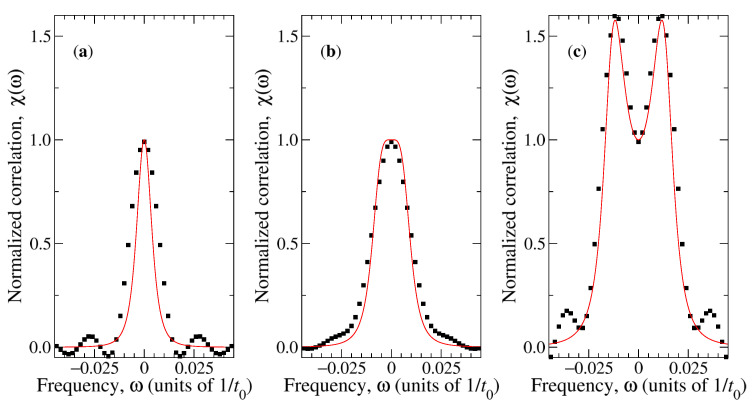
Autocorrelation function for the angular deviation with respect to the instantaneous magnetic field during rotation of magnetic capsules. (**a**) $\mu B=10\;k_{B}T$; (**b**) $\mu B=20\;k_{B}T$; (**c**) $\mu B=50\;k_{B}T$. Symbols correspond to simulation results and continuous curves of the estimation obtained from Equation (22).

Fluctuations around the average trajectory are analysed in terms of the normalised correlation χ(ω) defined by Equation (22). This correlation is calculated in simulations using the resulting time-series ϑ(t) assuming stationarity and applying a simple filtering process based on a moving average. Results for the specific case of $\omega_{0}=\pi \times 10^{-4}t_{0}^{-1}$ are compared with predictions of the model in Figure 4. Numerical correlations are well-approximated by the analytical curves for the three considered magnetic fields, which cover underdamped ($\mu B=50\;k_{B}T$) and overdamped dynamics ($\mu B=10\;k_{B}T$ and $\mu B=20\;k_{B}T$). The agreement is better in the case $\mu B=50\;k_{B}T$ (Figure 4c), while in cases $\mu B=10\;k_{B}T$ and $\mu B=20\;k_{B}T$ (Figure 4a,b, respectively), the analytical model slightly underestimates the actual width of the autocorrelation function. In addition, systematic oscillations can be identified at the tails of χ(ω) in the simulation case. Both of these effects are due to the fact that the stochastic process ϑ(t) is nonstationary but its autocorrelation has been estimated under the stationary approximation. Nevertheless, the good correspondence presented in Figure 4 provides confidence in using the proposed MD-N-MPCD method to simulate the nonequilibrium stochastic dynamics of the magnetic capsule.

### 3.3. Elastic Energy Changes and Topological Defect Deformations

The rotational Reynolds number and the Ericksen number are two dimensionless quantities that can be used to characterise the rotation of the capsule in the nematic liquid crystal. The former is defined as the ratio of the inertial torque to the viscous torque on the CP [60] and can be estimated as

(25) Re(r)=ρω0L2η,

where ρ is the solvent mass density. Then, it can be noticed that simulations were conducted with Re(r)=0.05 and Re(r)=0.1 for $\omega_{0}=\pi \times 10^{-4}t_{0}^{-1}$ and $\omega_{0}=2\pi \times 10^{-4}t_{0}^{-1}$, respectively. Er is the ratio of viscous-to-elastic effects and is customarily defined as

(26) $$\mathrm{Er}=\frac{\gamma_{1}v_{\mathrm{char}}l_{\mathrm{char}}}{K},$$

where $\gamma_{1}$ is a rotational viscosity and $v_{\mathrm{char}}$ ($l_{\mathrm{char}}$) is a characteristic velocity (length). The ratio $K/\gamma_{1}$ can be estimated from the spectrum of director fluctuations as discussed in References [55,61]. Using the same set of MPCD parameters as here but with $U=20\;k_{B}T$, it is obtained $K/\gamma_{1}≃0.62a^{2}t_{0}^{-1}$ [61]. However, this estimation is not expected to vary noticeably over a wide range of *U* values [55]. On the other hand, $l_{\mathrm{char}}$ and $v_{\mathrm{char}}$ can be taken from mapping the angular velocity of the CP into translational velocity, i.e., $l_{\mathrm{char}}∼L$ and $v_{\mathrm{char}}∼L\omega_{0}/2\pi$. Thus, Er∼0.032 and Er∼0.016 are estimated for simulations with $\omega_{0}=\pi \times 10^{-4}t_{0}^{-1}$ and $\omega_{0}=2\pi \times 10^{-4}t_{0}^{-1}$, respectively. This analysis permits situating simulations in the regime of small Reynolds and Ericksen numbers, where viscous effects dominate over inertial effects and elastic forces dominate over viscous forces.

**Figure 5:**
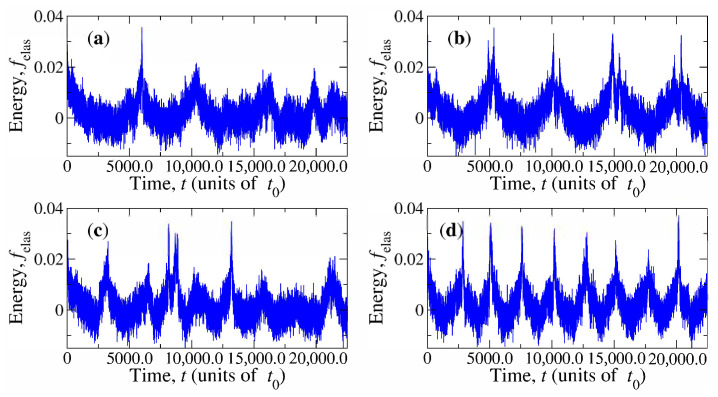
Normalised changes of the elastic energy of the nematic solvent as a consequence of the magnetic capsule rotation. The nematicity parameter is fixed at $U=6.5\;k_{B}T$. (**a**) $\omega_{0}=\pi \times 10^{-4}t_{0}^{-1}$ and $\mu B=10\;k_{B}T$; (**b**) $\omega_{0}=\pi \times 10^{-4}t_{0}^{-1}$ and $\mu B=50\;k_{B}T$; (**c**) $\omega_{0}=2\pi \times 10^{-4}t_{0}^{-1}$ and $\mu B=10\;k_{B}T$; (**d**) $\omega_{0}=2\pi \times 10^{-4}t_{0}^{-1}$ and $\mu B=50\;k_{B}T$.

**Figure 6:**
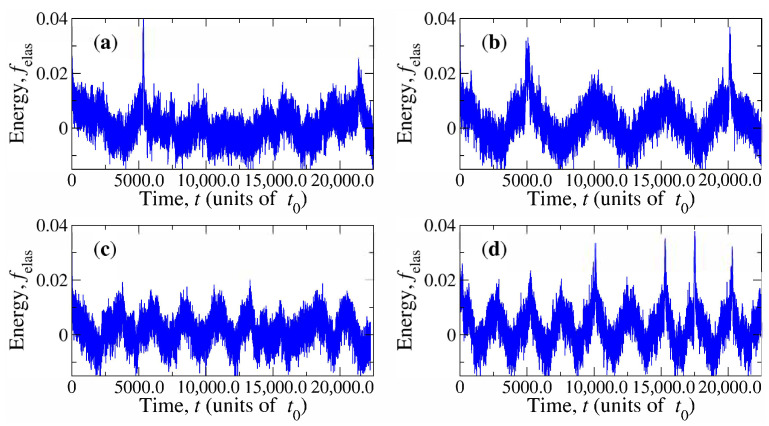
The same as in Figure 5 for $U=10\;k_{B}T$.

It is observed that $\hat{n}$ is periodically distorted as the capsule rotates due to the resulting moving anchoring imposed on its surface. The rotatory motion of the magnetic CP is accompanied by a corresponding rotation of the disclination ring. These effects can be studied in terms of time-dependent elastic energy $F_{\mathrm{elas}}\left(t\right)$, which will hereafter be simplified to the normalised form

(27) $$f_{\mathrm{elas}}\left(t\right)=\frac{F_{\mathrm{elas}}\left(t\right)-F_{\mathrm{elas}}^{\mathrm{eq}}}{F_{\mathrm{elas}}^{\mathrm{eq}}},$$

where $F_{\mathrm{elas}}^{\mathrm{eq}}$ is the equilibrium average elastic energy at B=0. The behaviour of $f_{\mathrm{elas}}\left(t\right)$ is illustrated in Figure 5 and Figure 6 for solvents with $U=6.5\;k_{B}T$ and $U=10\;k_{B}T$, respectively. For each nematicity, four results are presented for the combination of parameters $B=10\;k_{B}T$, $B=50\;k_{B}T$, $\omega_{0}=\pi \times 10^{-4}t_{0}^{-1}$, and $\omega_{0}=2\pi \times 10^{-4}t_{0}^{-1}$. Energy exhibits periodic increments. They appear with the frequency of the rotational motion of the capsule. These increments are, in general, stronger for larger magnetic fields and at some particular events, they have the shape of a remarkable peak that could be as large as 4% the elastic energy of the solvent in the simulation box. A comparison between Figure 5 and Figure 6 suggests that these large energy increments are more common for $U=6.5\;k_{B}T$.

**Figure 7:**
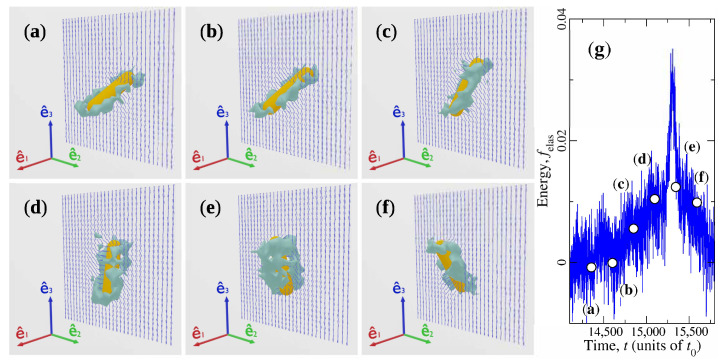
Morphological transformation of the topological defect around the rotating magnetic nanocapsule. (**a**–**f**) Successive configurations of the capsule and its companion defect as the former approaches and leaves behind parallel orientation with respect to ${\hat{n}}_{0}={\hat{e}}_{3}$. (**g**) Normalised elastic energy during the time interval in which transformation occurs. Energy and time associated with each configuration is represented by the labelled symbols in (**g**).

By inspecting the time evolution of the capsule, it can be concluded that $f_{\mathrm{elas}}$ increments appear when ${\hat{e}}_{3}^{\prime }$ gets close to $\pm {\hat{n}}_{0}$. At these states, noticeable transformations of the topological defect can take place. This process is illustrated in Figure 7 for the specific case of parameters $U=10\;k_{B}T$, $\omega_{0}=2\pi \times 10^{-4}t_{0}^{-1}$, and $\mu B=50\;k_{B}T$. Figure 7a–f are snapshots of the capsule–defect couple as it traverses the energy peak located in the time interval $t\in \;(14,250\;t_{0},15,750\;t_{0})$ in Figure 5. The CP rotates, and when its long symmetry axis coincides with ${\hat{e}}_{3}$, the form of the companion defect changes from a disclination ring to a broader structure that almost completely surrounds the cylindrical portion of the CP (Figure 7d,e). This transformation is reverted as capsule’s orientation departs from the director field at far distances. Supplementary Video S2 provides an animation of the capsule’s rotation and the topological defect changes for the same parameters used in Figure 7. Similar changes have been observed recently in defects around elongated particles driven by an external shear flow [36]. Here, their occurrence can be explained in terms of the orientation angle θ as follows. When θ≠0, the elongated, bent Saturn ring defect has been proven to be the structure with the smallest elastic energy [11,36,62]. However, for θ≃0, the most stable structure is a small ring located close to one of the ends of nanocapsule, while the disclination ring is a metastable configuration of larger elastic energy [11,36]. Due to the rotating magnetic field, the capsule–defect pair is driven to the state θ≃0, possessing large elastic energy. Then, the system attempts to reduce this energy by changing the shape of the defect from the unstable large ring to the small ring at the top or bottom of the CP. During this process, the region of small order spreads around the capsule. However, the transition to the small ring is frustrated because the continuously changing field removes the anisotropic CP from θ≃0, and the stability of the elongated Saturn ring is recovered.

**Figure 8:**
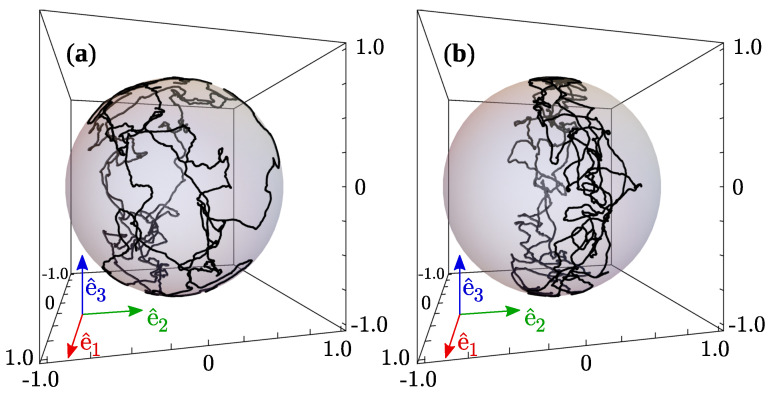
Orientation of the magnetic capsule over the unit sphere. (**a**) $\mu B=10\;k_{B}T$; (**b**) $\mu B=50\;k_{B}T$.

Interestingly, Figure 6c suggests that morphological transformations of the disclination rings can be eluded since elastic energy increments are kept moderate during the whole simulation. Indeed, no such transformations were observed for that case, corresponding to $U=10\;k_{B}T$, $\mu B=10\;k_{B}T$, and $\omega_{0}=2\pi \times 10^{-4}t_{0}^{-1}$. The reason is that, for small magnetic fields and large nematicity, it is expected that the torque exerted by the solvent on the CP will be able to act against the magnetic torque. Then, as the capsule–defect pair approaches the state θ=0 and the nematic torque tilts the CP out of the ${\hat{e}}_{1}-{\hat{e}}_{3}$ plane. This permits preserving the shape of the topological defect around the capsule. Figure 8a,b illustrate, respectively, the trajectories of the orientation vector ${\hat{e}}_{3}^{\prime }$ over the unit sphere obtained from simulations at $\left(U=10\;k_{B}T,\mu B=10\;k_{B}T\right)$ and $\left(U=10\;k_{B}T,\mu B=50\;k_{B}T\right)$. In the first case, the inclination about the ${\hat{e}}_{1}-{\hat{e}}_{3}$ plane is significant, whereas in the second one, the strength of the magnetic field maintains rotations of the capsule very close to that plane. By comparing trajectories in Figure 8a,b with the corresponding energy changes, Figure 7c,d, respectively, it can be concluded that the tilt induced by elastic torques prevents large energy peaks associated with defect shape transformations. This also explains why such peaks appear more frequently in the case $U=6.5\;k_{B}T$ (Figure 5) than in the case $U=10\;k_{B}T$ (Figure 6).

## 4. Discussion and Conclusions

Techniques from MD and N-MPCD were combined into a single algorithm with multiscale characteristicsthat recreated the dynamics of individualmagnetic nanocapsules immersed in nematic liquid crystals and subjected to external fields. The method shows that time-varying magnetic fields can be used to effectively control the rotation of the nanocapsules in the liquid crystal along with the topological defects around them. Capsules rotate at the pace of the magnetic field when the latter rotates at small frequencies. A phase retardation between magnetic field and capsule rotations could be observed, which was in agreement with predictions of a simplified dynamical model. The power spectrum of capsule orientation fluctuations along the main rotating trajectory was quantified and found to be in agreement with theoretical approximations. This encourages us to propose that MD-N-MPCD can be used in complex nonequilibrium situations involving the interaction of liquid crystals with moving objects. Consequently, it could be particularly well-suited for simulating particles in anisotropic fluids where external forces cause translational and rotational motion of the immersed particles. It could also be used to study the self-assembly process of anisotropic CPs or the dynamics of active CPs in liquid crystals.

During the rotation of the anisotropic CP, the associated topological defect suffers morphological changes and a consequential elastic energy gain is observed in the solvent. These changes appear as a response of the solvent to the action of the magnetic field that drives the capsule–defect pair to an orientational state of high elastic energy. Then, a reorientation process of the director field around the CP occurs that intends to transform the disclination ring into a defect of smaller elastic energy. However, such transformation is prevented by the magnetic field itself that drives the CP back to weak nonequilibrium states where the disclination defect is more convenient again. This nonequilibrium effect is similar to that observed for anisotropic particles rotating in a sheared nematic [36]. However, it should be noticed that energy gains sustained by magnetically induced colloidal rotations avoid global nematic flow and, therefore, they could be easier to produce in practice. Furthermore, the aforementioned morphological changes are obtained for rotational Reynolds numbers Re(r)∼0.05 and Ericksen numbers $\mathrm{Er}∼10^{-2}$, which are smaller than those considered in the nonequilibrium situation induced by shear. This suggests that they could be experimentally accessible.

When magnetic torques were moderate compared with torques exerted by the nematic solvent, morphological transformations of defects were not observed. This is because the elastic torque on the capsule counteracts the magnetic torque and prevents the system from being driven far from equilibrium. This result is in very good qualitative agreement with recent experimental observations in which peanut-shaped dipolar CPs were rotated by magnetic fields and tilted out of the rotation plane by the nematic liquid crystal [16]. All this reinforces the reliability of MD-N-MPCD as a promising method to deal with nonequilibrium dynamics in liquid crystal colloids.

## Figures and Tables

**Table 1:** Units and parameters used for MD-N-MPCD simulations. All these parameters must be given independently to specify a particular numerical execution.

Derived Units	Fluid Parameters	Colloid Parameters
$t_{0}=a\sqrt{m/k_{B}T}$	${\bar{N}}^{c}=20$	$\delta \;t=0.01\;t_{0}$
$\eta_{0}=\sqrt{mk_{B}T}/a^{2}$	$\Delta \;t=1\;t_{0}$	L=10a
	$L_{1}=L_{2}=L_{3}=64\;a$	σ=2a
	U=6.5 and $10\;k_{B}T$	$\epsilon =10\;k_{B}T$
	λ=1.2	$\rho_{\mathrm{col}}=100\;m/a^{3}$
	$\chi_{\mathrm{HI}}=1.0$	$\sigma_{S}=15/a^{2}$
	$\gamma_{R}=0.01\;a^{2}\eta_{0}$	

## Data Availability

Not applicable.

## Supplementary Materials

The following are available online at https://www.mdpi.com/article/10.3390/ma14112886/s1. Video S1: Brownian motion of the nematic capsule at B=0. Video S2: rotation of the nematic capsule at $U=10\;k_{B}T$, $B=50\;k_{B}T$, and $\omega_{0}=2\pi \times 10^{-4}t_{0}^{-1}$.

## Abbreviations

The following abbreviations are used in this manuscript:

CP	Colloidal particle
MD	Molecular Dynamics
N-MPCD	Nematic Multiparticle Collision Dynamics

## Appendix A

Under the assumption of two-dimensional rotation, one has $\theta \;=\omega_{0}t+\vartheta$. This allows us to write Equation (19) in terms of ϑ as

(A1) $$J_{1}\ddot{\vartheta }=-\eta_{R}\left(\dot{\vartheta }+\omega_{0}\right)-\mu B\sin\vartheta +\Delta \;F\sin\left(2\vartheta \;+2\omega_{0}t\right)\;+\;\tau \left(t\right).$$

Equation (A1) does not admit an analytical solution due to its nonlinear character. However, in the limit of large magnetic torques ΔF/μB≪1, capsules are expected to follow the magnetic field closely and ϑ could be considered small, i.e., ϑ≪1. By expanding Equation (A1) in terms of the previously defined small quantities and retaining only linear terms, one has

(A2) $$\ddot{\vartheta }=-\beta \;\dot{\vartheta }-\omega_{B}^{2}\vartheta \;+f\left(t\right)+\tilde{\tau }\left(t\right),$$

where $\beta \;=\eta_{R}/J_{1}$, $\omega_{B}^{2}=\mu B/J_{1}$, $f\left(t\right)=-\beta \;\omega_{0}+\Delta \;F\sin\left(2\omega_{0}t\right)/J_{1}$, and $\tilde{\tau }\left(t\right)=\tau \left(t\right)/J_{1}$.

The solution of Equation (A2) is

(A3) $$\vartheta \left(t\right)=\phi \left(t\right)\vartheta_{0}+\psi \left(t\right){\dot{\vartheta }}_{0}+g\left(t\right)+\int_{0}^{t}dt^{\prime }\psi \left(t-t^{\prime }\right)\tilde{\tau }\left(t^{\prime }\right),$$

where $\vartheta_{0}=\vartheta \left(0\right)$ and ${\dot{\vartheta }}_{0}=\dot{\vartheta }\left(0\right)$, and ϕ(t) and ψ(t) are auxiliary functions defined by

(A4) $$\phi \left(t\right)=\frac{\mu_{2}}{\mu_{2}-\mu_{1}}e^{\mu_{1}t}+\frac{\mu_{1}}{\mu_{1}-\mu_{2}}e^{\mu_{2}t},$$

(A5) $$\psi \left(t\right)=\frac{1}{\mu_{1}-\mu_{2}}e^{\mu_{1}t}+\frac{1}{\mu_{2}-\mu_{1}}e^{\mu_{2}t},$$

with

(A6) $$\mu_{1,2}=-\frac{\beta }{2}\pm \sqrt{\frac{\beta^{2}}{4}-\omega_{B}^{2}}.$$

Function g(t) in Equation (A6) is an abbreviation for

(A7) $$\begin{array}{ccc} g(t) & = & \int_{0}^{t}dt^{\prime }\psi \left(t-t^{\prime }\right)f\left(t^{\prime }\right)\\ & = & \frac{\beta \;\omega_{0}}{\mu_{1}-\mu_{2}}\left(\frac{1-e^{\mu_{1}t}}{\mu_{1}}-\frac{1-e^{\mu_{2}t}}{\mu_{2}}\right)+\frac{\Delta \;F}{2J_{1}\omega_{0}\left(\mu_{1}-\mu_{2}\right)}\\ & & \times \left[e^{\mu_{1}t}\sin^{2}\alpha_{1}-e^{\mu_{2}t}\sin^{2}\alpha_{2}+\sin\left(\alpha_{2}-\alpha_{1}\right)\sin\left(2\omega_{0}t+\alpha_{1}+\alpha_{2}\right)\right], \end{array}$$

where $\tan\alpha_{1,2}=2\omega_{0}/\mu_{1,2}$.

Equation (21) is obtained from the average of the stationary limit of ϑ(t),

(A8) $$\begin{array}{c} \theta_{\mathrm{stat}}=\omega_{0}t+\lim_{t\beta \;\gg 1}\langle \vartheta \;\left(t\right)\rangle \end{array}$$

and using the definitions of β, $\omega_{B}^{2}$, $\mu_{1,2}$, and $\alpha_{1,2}$.

To calculate the autocorrelation function of ϑ, a further simplification is made by neglecting the nematic torque and retardation in Equation (A2). The solution of the resulting expression in Fourier domain reads as

(A9) $$\vartheta \left(\omega \right)=\frac{\tilde{\tau }\left(\omega \right)}{\left(i\omega \;-\mu_{1}\right)\left(i\omega \;-\mu_{2}\right)},$$

with $i^{2}=-1$. Then, by multiplying ϑ(ω) by $\vartheta (\omega^{\prime })$, and using the Fourier transform of Equation (20), namely,

(A10) $$\langle \tau \left(\omega^{\prime }\right)\tau \left(\omega \right)\rangle =2\left(2\pi \right)k_{B}T\eta_{R}\delta \left(\omega \;+\omega^{\prime }\right),$$

it follows that

(A11) $$\langle \vartheta \left(\omega \right)\vartheta^{*}\left(\omega \right)\rangle =\frac{2\left(2\pi \right)k_{B}T\eta_{R}\delta \left(0\right)}{\left(i\omega \;-\mu_{1}\right)\left(i\omega \;-\mu_{2}\right)\left(-i\omega \;-\mu_{1}^{*}\right)\left(-i\omega \;-\mu_{2}^{*}\right)}.$$

Equation (22) follows after substituting Equation (A6) into Equation (A11) and normalising.

## References

[1] De Gennes P.G., Prost J. (1993). The Physics of Liquid Crystals.

[2] Muševič I. (2017). Liquid Crystal Colloids.

[3] Muševič I. (2018). Nematic Liquid-Crystal Colloids. Materials.

[4] Kim Y.K., Noh J., Nayani K., Abbott N.L. (2018). Soft matter from liquid crystals. Soft Matter.

[5] Stark H. (2011). Physics of colloidal dispersions in nematic liquid crystals. Phys. Rep..

[6] Kleman M., Lavrentovich O.D. (2003). Soft Matter Physics: An Introduction.

[7] Smalyukh I.I. (2018). Liquid Crystal Colloids. Annu. Rev. Condens. Matter Phys..

[8] Ravnik M., Žumer S. (2009). Nematic colloids entangled by topological defects. Soft Matter.

[9] Poulin P., Weitz D.A. (1998). In verted and multiple nematic emulsions. Phys. Rev. E.

[10] Loudet J.C., Poulin P. (2001). Application of an Electric Field to Colloidal Particles Suspended in a Liquid-Crystal Solvent. Phys. Rev. Lett..

[11] Hung F.R., Guzmán O., Gettelfinger B.T., Abbott N.L., de Pablo J.J. (2006). Anisotropic nanoparticles immersed in a nematic liquid crystal: Defect structures and potentials of mean force. Phys. Rev. E.

[12] Hung F.R. (2009). Quadrupolar particles in a nematic liquid crystal: Effects of particle size and shape. Phys. Rev. E.

[13] Tasinkevych M., Loudet J., Mondain O., Mondiot F. (2013). Dispersions of ellipsoidal particles in a nematic liquid crystal. Soft Matter.

[14] Hashemi S.M., Ejtehadi M.R. (2015). Equilibrium state of a cylindrical particle with flat ends in nematic liquid crystals. Phys. Rev. E.

[15] Gharbi M.A., Cavallaro M., Wu G., Beller D.A., Kamien R.D., Yang S., Stebe K.J. (2013). Microbullet assembly: Interactions of oriented dipoles in confined nematic liquid crystal. Liq. Cryst..

[16] Sahu D.K., Anjali T.G., Basavaraj M.G., Aplinc J., Čopar S., Dhara S. (2019). Orientation, elastic interaction and magnetic response of asymmetric colloids in a nematic liquid crystal. Sci. Rep..

[17] Lapointe C.P., Mason T.G., Smalyukh I.I. (2009). Shape-Controlled Colloidal Interactions in Nematic Liquid Crystals. Science.

[18] Liu Q., Senyuk B., Tasinkevych M., Smalyukh I.I. (2013). Nematic liquid crystal boojums with handles on colloidal handlebodies. PNAS.

[19] Senyuk B., Liu Q., He S., Kamien R.D., Kusner R.B., Lubensky T.C., Smalyukh I.I. (2013). Topological colloids. Nature.

[20] Hashemi S.M., Jagodič U., Mozaffari M.R., Ejtehadi M.R., Muševič I., Ravnik M. (2017). Fractal nematic colloids. Nat. Commun..

[21] Völtz C., Maeda Y., Tabe Y., Yokoyama H. (2006). Director-Configurational Transitions around Microbubbles of Hydrostatically Regulated Size in Liquid Crystals. Phys. Rev. Lett..

[22] Gu Y., Abbott N.L. (2000). Observation of Saturn-Ring Defects around Solid Microspheres in Nematic Liquid Crystals. Phys. Rev. Lett..

[23] Yoneya M., Fukuda J.I., Yokoyama H., Stark H. (2005). Effect of a Hydrodynamic Flow on the Orientation Profiles of a Nematic Liquid Crystal Around a Spherical Particle. Mol. Cryst. Liq. Cryst..

[24] Araki T., Tanaka H. (2006). Surface-sensitive particle selection by driving particles in a nematic solvent. J. Phys. Condens. Matter.

[25] Khullar S., Zhou C., Feng J.J. (2007). Dynamic Evolution of Topological Defects around Drops and Bubbles Rising in a Nematic Liquid Crystal. Phys. Rev. Lett..

[26] Zhou C., Yue P., Feng J.J. (2007). The rise of Newtonian drops in a nematic liquid crystal. J. Fluid Mech..

[27] Stieger T., Schoen M., Mazza M.G. (2014). Effects of flow on topological defects in a nematic liquid crystal near a colloid. J. Chem. Phys..

[28] Stieger T., Püschel-Schlotthauer S., Schoen M., Mazza M.G. (2016). Flow-induced deformation of closed disclination lines near a spherical colloid immersed in a nematic host phase. Mol. Phys..

[29] Reyes-Arango D., Quintana H.J., Armas-Pérez J.C., Híjar H. (2020). Defects around nanocolloids in nematic solvents simulated by Multi-particle Collision Dynamics. Phys. A.

[30] Villada-Gil S., Palacio-Betancur V., Armas-Pérez J.C., de Pablo J.J., Hernández-Ortiz J.P. (2021). Directing the far-from-equilibrium assembly of nanoparticles in confined liquid crystals by hydrodynamic fields. Soft Matter.

[31] Cavallaro M., Gharbi M.A., Beller D.A., Čopar S., Shi Z., Baumgart T., Yang S., Kamien R.D., Stebe K.J. (2013). Exploiting imperfections in the bulk to direct assembly of surface colloids. Proc. Natl. Acad. Sci. USA.

[32] Lee E., Xia Y., Ferrier R.C., Kim H.N., Gharbi M.A., Stebe K.J., Kamien R.D., Composto R.J., Yang S. (2016). Fine Golden Rings: Tunable Surface Plasmon Resonance from Assembled Nanorods in Topological Defects of Liquid Crystals. Adv. Mater..

[33] Peng C., Turiv T., Guo Y., Shiyanovskii S.V., Wei Q.H., Lavrentovich O.D. (2016). Control of colloidal placement by modulated molecular orientation in nematic cells. Sci. Adv..

[34] Luo Y., Beller D.A., Boniello G., Serra F., Stebe K.J. (2018). Tunable colloid trajectories in nematic liquid crystals near wavy walls. Nat. Commun..

[35] Luo Y., Yao T., Beller D.A., Serra F., Stebe K.J. (2019). Deck the Walls with Anisotropic Colloids in Nematic Liquid Crystals. Langmuir.

[36] Híjar H. (2020). Dynamics of defects around anisotropic particles in nematic liquid crystals under shear. Phys. Rev. E.

[37] Muševič I., Škarabot M., Babič D., Osterman N., Poberaj I., Nazarenko V., Nych A. (2004). Laser Trapping of Small Colloidal Particles in a Nematic Liquid Crystal: Clouds and Ghosts. Phys. Rev. Lett..

[38] Lucchetti L., Criante L., Bracalente F., Aieta F., Simoni F. (2011). Optical trapping induced by reorientational nonlocal effects in nematic liquid crystals. Phys. Rev. E.

[39] Chen S.H., Amer N.M. (1983). Observation of Macroscopic Collective Behavior and New Texture in Magnetically Doped Liquid Crystals. Phys. Rev. Lett..

[40] Lapointe C., Cappallo N., Reich D.H., Leheny R.L. (2005). Static and dynamic properties of magnetic nanowires in nematic fluids. J. Appl. Phys..

[41] Lapointe C., Hultgren A., Silevitch D.M., Felton E.J., Reich D.H., Leheny R.L. (2004). Elastic Torque and teh Levitation of Metal Wires by a Nematic Liquid Crystal. Science.

[42] Varney M.C.M., Jenness N.J., Smalyukh I.I. (2014). Geometrically unrestricted, topologically constrained control of liquid crystal defects using simultaneous holonomic magnetic and holographic optical manipulation. Phys. Rev. E.

[43] Cirtoaje C., Petrescu E. (2019). The Influence of Single-Walled Carbon Nanotubes on the Dynamic Properties of Nematic Liquid Crystals in Magnetic Field. Materials.

[44] Armendáriz J., Híjar H. (2021). Magnetic Anisotropic Colloids in Nematic Liquid Crystals: Fluctuating Dynamics Simulated by Multi-particle Collision Dynamics. Int. J. Mod. Phys. B.

[45] Allen M.P., Tildesley D.J. (2017). Computer Simulation of Liquids.

[46] Frenkel D., Smit B. (2002). Understanding Molecular Simulation.

[47] Omelyan I.P. (1998). Numerical integration of the equations of motion for rigid polyatomics: The matrix method. Comput. Phys. Commun..

[48] Gompper G., Ihle T., Kroll D.M., Winkler R.G., Holm C., Kremer K. (2009). Multi-Particle Collision Dynamics—A Particle-Based Mesoscale Simulation Approach to the Hydrodynamics of Complex Fluids. Advanced Computer Simulation Approaches for Soft Matter Sciences III.

[49] Padding J.T., Louis A.A. (2006). Hydrodynamic interactions and Brownian forces in colloidal suspensions: Coarse-graining over time and length scales. Phys. Rev. E.

[50] Lee K.W., Mazza M.G. (2015). Stochastic rotation dynamics for nematic liquid crystals. J. Chem. Phys..

[51] Shendruk T.N., Yeomans J.M. (2015). Multi-particle collision dynamics algorithm for nematic fluids. Soft Matter.

[52] Mandal S., Mazza M.G. (2019). Multiparticle Collision Dynamics for Tensorial Nematodynamics. Phys. Rev. E.

[53] Osipov M.A., Demus D., Goodby J., Gray G.W., Spiess H.-W., Vill V. (1998). Molecular Theories of Liquid Crystals. Handbook of Liquid Crystals.

[54] Jeffery G.B. (1922). The motion of ellipsoidal particles immersed in a viscous fluid. Proc. R. Soc. Lond. A.

[55] Híjar H., Halver R., Sutmann G. (2019). Spontaneous Fluctuations in Mesoscopic Simulations of Nematic Liquid Crystals. Fluct. Noise Lett..

[56] Ihle T., Kroll D.M. (2001). Stochastic rotation dynamics: A Galilean-invariant mesoscopic model for fluid flow. Phys. Rev. E.

[57] Whitmer J.K., Luijten E. (2010). Fluid–solid boundary conditions for multiparticle collision dynamics. J. Phys. Condens. Matter.

[58] Burylov S.V., Raikher Y.L. (1994). Orientation of a solid particle embedded in a monodomain nematic liquid crystal. Phys. Rev. E.

[59] Dhont J.K.G. (1996). An Introduction to Dynamics of Colloids.

[60] Happel J., Brenner H. (1983). Low Reynolds Number Hydrodynamics.

[61] Híjar H. (2019). Hydrodynamic correlations in isotropic fluids and liquid crystals simulated by multi-particle collision dynamics. Condens. Matter Phys..

[62] Andrienko D., Allen M.P., Skačej G., Žumer S. (2002). Defect structures and torque on an elongated colloidal particle immersed in a liquid crystal host. Phys. Rev. E.

