# Supplementary figures and images for: Nonequilibrium Dynamics of a Magnetic Nanocapsule in a Nematic Liquid Crystal
Source: Materials (Basel). 2021 May 27;14(11):2886. doi: 10.3390/ma14112886 (PMC8199132; doi:10.3390/ma14112886)

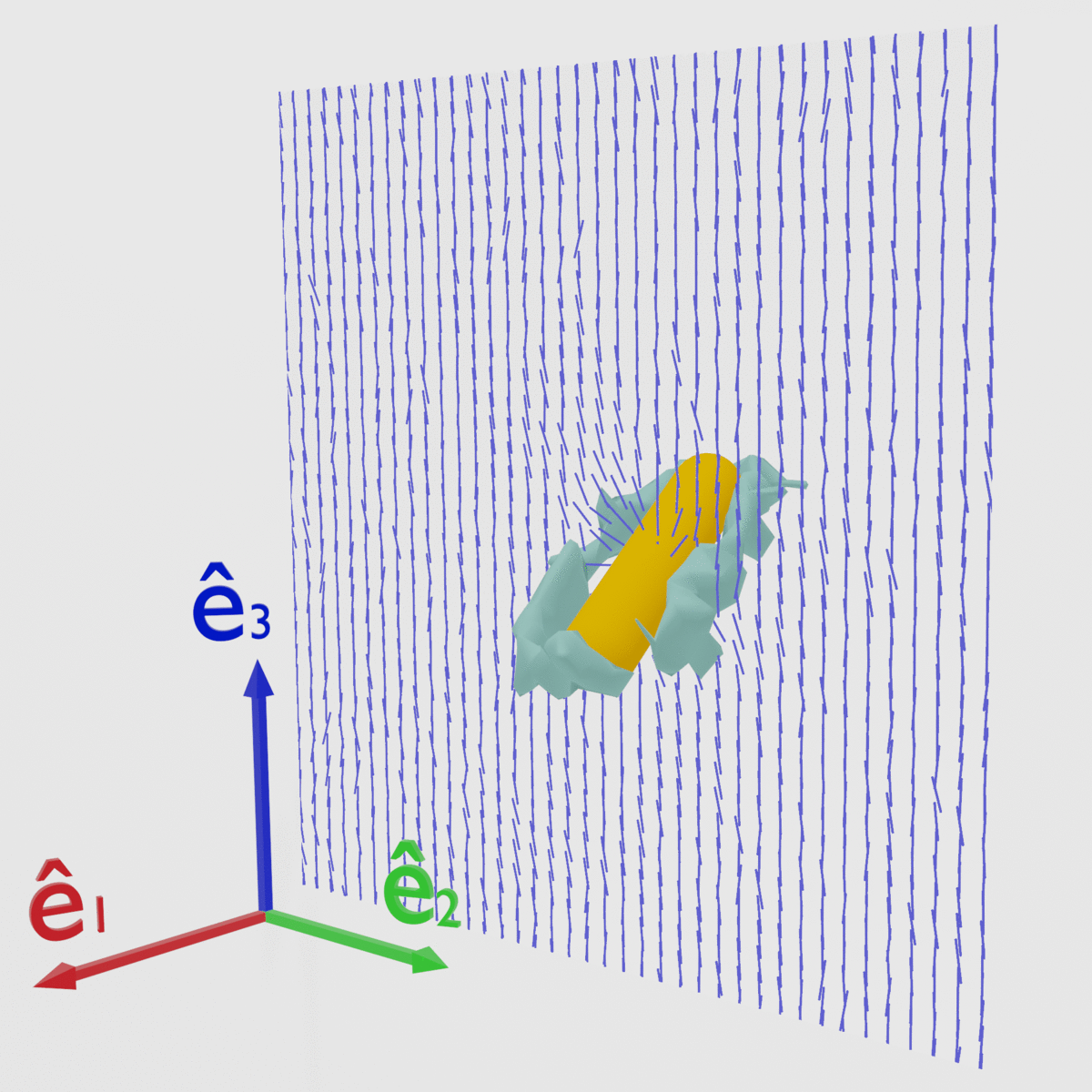

Supplement: Supplementary file 1 [file materials-14-02886-s001.zip › video_s1.gif]

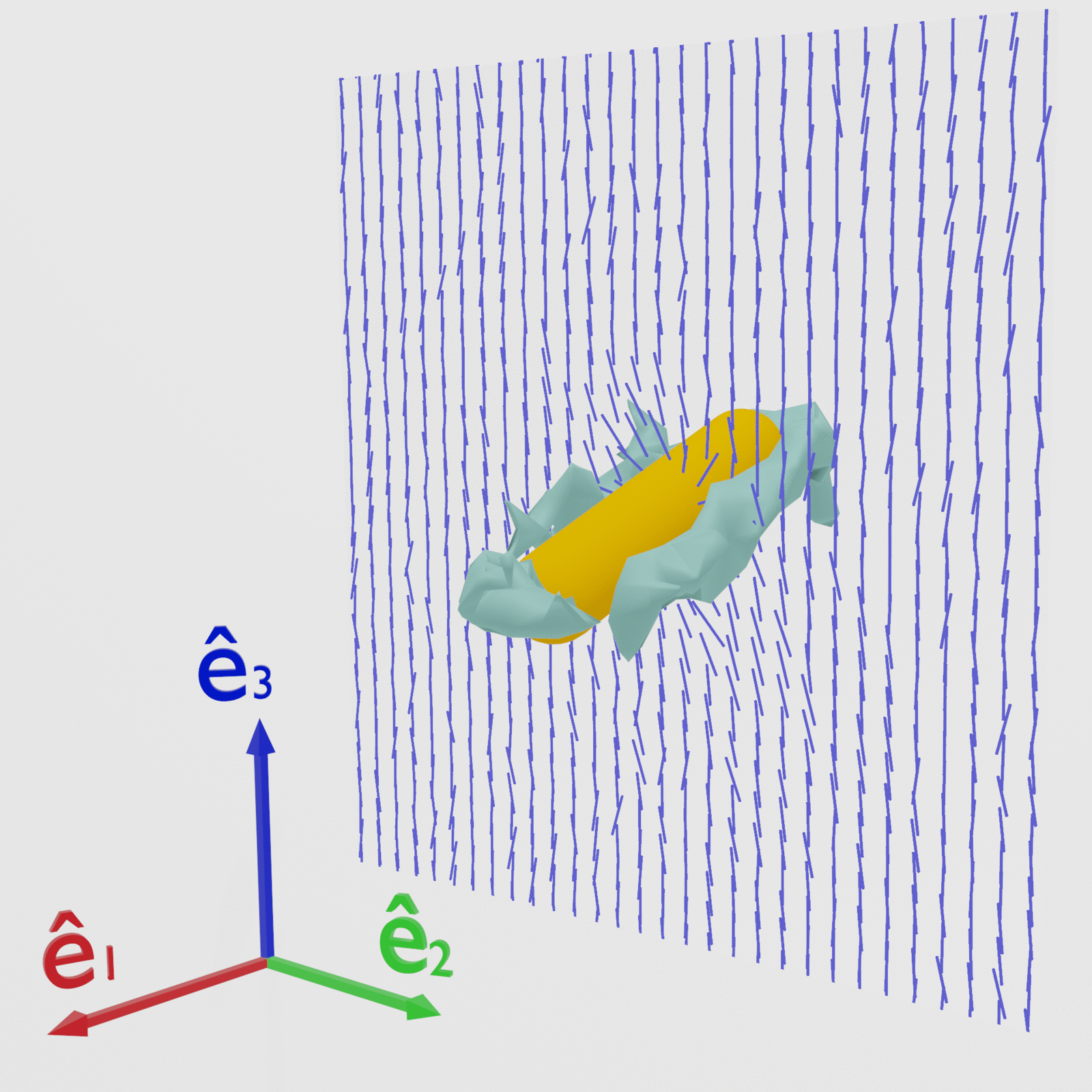

Supplement: Supplementary file 1 [file materials-14-02886-s001.zip › video_s2.gif]
